# An Explainable Deep-Learning Approach to Detect Pediatric Sleep Apnea From Single-Channel Airflow

**DOI:** 10.1109/JTEHM.2025.3625388

**Published:** 2025-10-24

**Authors:** Verónica Barroso-García, Fernando Vaquerizo-Villar, Gonzalo C. Gutiérrez-Tobal, Ehab Dayyat, David Gozal, Timo Leppänen, Roberto Hornero

**Affiliations:** Biomedical Engineering GroupUniversidad de Valladolid Valladolid 47011 Spain; Centro de Investigación Biomédica en Red en BioingenieríaBiomateriales y Nanomedicina (CIBER-BBN) Madrid Spain; Department of Pediatric Neurology and Sleep MedicineBaylor Scott and White McLane Children’s Medical Center Temple TX 76502 USA; Department of PediatricsJoan C. Edwards School of MedicineMarshall University 4034 Huntington WV 25701 USA; Department of Technical PhysicsUniversity of Eastern Finland 163043 Kuopio 70211 Finland; Diagnostic Imaging CenterKuopio University Hospital 60650 Kuopio 70210 Finland; School of Electrical Engineering and Computer ScienceThe University of Queensland 1974 Brisbane QLD 4072 Australia

**Keywords:** Airflow, children, convolutional neural network (CNN), deep-learning (DL), explainable artificial intelligence (XAI), obstructive sleep apnea (OSA)

## Abstract

Objective: Approaches based on a single-channel airflow has shown great potential for simplifying pediatric obstructive sleep apnea (OSA) diagnosis. However, analysis has been limited to feature-engineering techniques, restricting identification of complex respiratory patterns, and reducing diagnostic performance in automated models. Here, we propose deep-learning and explainable artificial intelligence (XAI) to estimate the pediatric OSA severity from airflow, while ensuring transparency in automatic decisions. Technology or Method: We used 3,672 overnight airflow recordings from four pediatric datasets. A convolutional neural network (CNN)-based regression model was trained to estimate the apnea-hypopnea index (AHI) and predict OSA severity. We evaluated and compared Gradient-Weighted Class Activation Mapping (Grad-CAM) and SHapley Additive exPlanations (SHAP) to identify the airflow regions where the CNN focuses for predictions. Results: The proposed model demonstrated high concordance between the actual and estimated AHI (intraclass correlation coefficient from 0.69 to 0.87 in the test group), and high diagnostic performance: four-class Cohen’s kappa between 0.37 and 0.43 and accuracies of 82.03%, 97.09%, and 99.03% for three OSA severity cutoffs (i.e. 1, 5, and 10 e/h) in the test group. The interpretability analysis with Grad-CAM and SHAP revealed that the CNN accurately identifies apneic events by focusing on their onset and offset. Both techniques provided complementary information about the model’s decision-making. While Grad-CAM highlighted respiratory events with abrupt signal changes, SHAP captured more subtle patterns with noise included. Conclusions: Accordingly, our model can help automatically detect pediatric OSA and offers clinicians an explainable approach that enhances credibility and usability, thus providing a path toward clinical translation in early diagnosis. Clinical Impact: This study presents an interpretable deep-learning tool using airflow to accurately detect pediatric obstructive sleep apnea, enabling early, objective diagnosis and supporting clinical decision-making through identification of relevant respiratory patterns.

## I. Introduction

Pediatric sleep disordered breathing is frequent condition with its most serious form, obstructive sleep apnea (OSA) being estimated to affect between 2% and 4% of all children [1], [2]. However, the prevalence may be significantly higher in certain population groups, such as children with obesity or those with maxillofacial and/or craniofacial abnormalities [3], [4]. OSA-affected children and adolescents suffer from either significant reduction in airflow (hypopneas) or total breathing cessation (apnea) while they sleep [5], which trigger a wide range of adverse physiological alterations such as hypoxia and sleep fragmentation. These events evoke a complex cascade of pathophysiological pathways potentially resulting in serious comorbidities. Some of the most notable adverse outcomes include cardiovascular and metabolic dysfunction, growth and development alterations, behavioral and learning issues, as well as neurocognitive impairments [6], [7], [8]. Therefore, early detection and intervention of OSA are crucial since if not treated in a timely manner, OSA can produce long-lasting negative consequences on both the health and well-being of affected children.

The gold standard method for OSA diagnosis is the in-lab nocturnal polysomnography (PSG), a specialized medical study that allows the assessment of sleep quality and a variety of physiological features throughout the night [9]. The key signals recorded during PSG include electroencephalogram (EEG), electrooculogram (EOG), electromyogram (EMG), electrocardiogram (ECG), blood oxygen saturation (SpO2), airflow, abdominal and thoracic movements, leg movement, and snoring monitoring, among others. Once these physiological signals have been recorded, the PSG record undergoes a laborious and time-consuming manual analysis and interpretation process by a sleep medicine specialist. The first step involves manually examining the data collected during PSG. Then, airflow reduction (30%-90% from airflow baseline) or cessation ( $\ge90$% from airflow baseline) episodes, blood oxygen saturation drops ( $\ge3$% from SpO2 baseline), and other OSA hallmark indicators are scored to obtain the apnea-hypopnea index (AHI) [5], [10]. This index, computed as the average number of apneic events per hour of sleep (e/h), is clinically used to classify patients to different OSA severity categories. It should be noted that PSG is conducted in a sleep laboratory involving specialized staff and equipment, and thus PSG is an onerous procedure [11]. Moreover, PSG is uncomfortable for children, potentially affecting their natural sleep quality and patterns [9] and PSG-derived measures reflect only the specific, single-night when it is conducted [12]. Finally, availability of PSG is limited in many countries and rural areas, which delays both diagnosis and treatment access [13].

To address these limitations, alternative diagnostic strategies have been explored. In this regard, analyzing cardiorespiratory signals, such as airflow, can provide unique opportunities for the automatic diagnosis of pediatric OSA [14]. Airflow could be easily acquired at patient’s home with an oronasal thermistor integrated into a portable monitoring device attached to children [12]. Moreover, airflow includes information on specific breathing patterns, such as the duration and frequency of apneas and hypopneas [5]. However, its automatic analysis in the context of pediatric OSA has been restricted to the use of feature-engineering (FE) techniques assessed independently from other signals [15], [16], [17], [18], [19]. These methods have proven remarkable effectiveness characterizing airflow changes in time and frequency domains during apneic events [15], [16], [17], [18], [19]. However, these approaches require a comprehensive prior knowledge of the features to be extracted from airflow data, which can become a complex and thorough task [20]. A deep understanding of the techniques to be applied and their suitability to adapt to the intrinsic properties of the overnight airflow is also needed. An additional challenge is that FE methods often focus on specific approaches, such as nonlinear or spectral analysis, which may overlook OSA-related information present in the airflow signal [20]. As a result, there is a risk of missing relevant information that could be crucial for OSA diagnosis comprehensive understanding of its determinants.

In contrast, approaches based on deep-learning (DL) could offer a promising solution to the challenges posed by traditional FE in single-channel airflow analysis. These methods are able to automatically uncover meaningful attributes and underlying patterns within the raw data, circumventing the need for hand-designed features [20], [21]. Moreover, DL methods are highly adaptable and versatile, making them well-suited for addressing key aspects of airflow signal analysis. They can efficiently capture both time and frequency domain information without confining themselves to specific approaches (explicit feature- or rule-based algorithms) [20], [21], allowing a more comprehensive analysis of the signal characteristics during apneic events. Accordingly, the individual or combined use of DL models based on convolutional neural networks (CNN) has shown an enhanced performance in diagnosing childhood OSA from cardiorespiratory signals when compared to conventional FE approaches [22], [23], [24], [25], [26]. However, we are unaware of any studies that analyze the single-channel airflow signal using DL. It should be noted that the DL models are capable of automatically identifying complex physiological OSA-derived patterns from raw biosignals [21], [24], but their interpretability (i.e., understanding the reasons behind decisions) and transparency (i.e., clarity of how predictions are made) are limited [27]. This loss of interpretability is a significant challenge, particularly in the medical field, where confidence and understanding of results are crucial for clinical decision-making. Healthcare professionals and physicians need to understand how a DL model reaches conclusions to trust its accuracy and make personalized and effective decisions regarding childhood OSA diagnosis and treatment.

To address this limitation and ensure the reliability of CNN models that automatically analyze the airflow signal, this study proposes the application of CNN along with eXplainable Artificial Intelligence (XAI) techniques. The goal of XAI is to offer a clear and comprehensible insight into the inner workings of the model and the rationale behind its decisions [28], [29]. In order to do this, these techniques pinpoint the regions of the signal that have a significant impact on the model’s decision-making [27], [28]. More specifically, we evaluated the Gradient-weighted Class Activation Mapping (Grad-CAM) and SHapley Additive explanation (SHAP) methods given their success in providing interpretability to CNN models [25], [27], [28], [30]. Although they are widely used XAI techniques, to the best of our knowledge, no OSA research exists that jointly applies them to single-channel airflow signals. Thus, our study began with the hypothesis that an interpretable DL model can automatically identify OSA respiratory patterns and detect its associated particularities in airflow among pediatric population. Beyond the methodological development, the primary aim of this work is to facilitate the clinical translation of artificial intelligence into pediatric sleep medicine. By providing an interpretable DL model, our approach aims to support early screening programs and clinical decision-making in real-world healthcare settings. Accordingly, the objectives were (i) to assess the usefulness of a CNN model for determining the severity of pediatric OSA from overnight airflow, and (ii) to pinpoint the regions of airflow signal on which the CNN focuses to make its prediction.

The main contributions of our study are the following:

• Design, implement, and validate a CNN model for automatically detecting complex respiratory patterns associated with pediatric OSA from airflow signal. To our knowledge, this is the first study that evaluated raw pediatric single-channel airflow signals from 4 large polysomnographic datasets using DL techniques.
• Provide interpretability, transparency, and reliability to an airflow-fed CNN model by jointly applying Grad-CAM and SHAP in the context of pediatric OSA. To the best of our knowledge, this is the first study that has jointly used both techniques to thoroughly understand which regions of airflow signal are crucial for diagnosing the disease.

## II. Pediatric Datasets and Airflow Signals

Our investigation involved 3,672 polysomnographic recordings from the following 4 pediatric datasets:

1) The Childhood Adenotonsillectomy Trial (CHAT, ClinicalTrials.gov Identifier: NCT00560859) [31], [32], a public and multicenter dataset composed of 1,638 overnight airflow recordings.
2) The Cleveland Children’s Sleep and Health Study (CCSHS) [32], [33], a public dataset from 3 Cleveland area hospitals composed of 515 airflow recordings.
3) A private dataset from the Comer Children’s Hospital of the University of Chicago (UofC). This dataset includes 974 airflow recordings.
4) A private dataset from the Le Bonheur Children’s Hospital of the University of Tennessee (UofT), consisting of 545 airflow recordings.

Informed consent was obtained from all caretakers as part of the study protocols of CHAT (defined in Marcus et al. [31]), CCSHS (established in Rosen et al. [33]), the Comer Children’s Hospital (#11-0268-AM017, #09-115-B-AM031, and #IRB14-1241, respectively), and the Le Bonheur Children’s Hospital (TN 16-04774-XM), which were approved by the Ethics Committee of participating medical centers. All children were diagnosed for OSA according to the apnea and hypopnea scoring criteria established by the American Academy of Sleep Medicine (AASM): the 2007 guidelines for CHAT and CCSHS (considering the variable ahi_a0h3a as the actual AHI), and the 2012 update for UofC and UofT [5], [34]. From the PSG-derived AHI, the specialist physicians determined whether these subjects were free of OSA (1 e/h $< $ AHI), or if they suffered from mild (1 e/h $\le$ AHI $< $ 5 e/h), moderate (5 e/h $\le$ AHI $< $ 10 e/h), or severe (AHI $\ge$ 10 e/h) OSA [8].

Global differences across cohorts were assessed using the Kruskal–Wallis test for continuous variables and the Chi-square test of independence for categorical variables. For post-hoc pairwise comparisons, the Mann–Whitney U test was applied to continuous variables and the Fisher test to categorical variables. To control type I error arising from multiple comparisons, Bonferroni correction was applied (adjusted $\alpha =$ 0.05/6 $\approx$ 0.0083). In this regard, statistically significant differences were observed in sociodemographic and clinical variables (see Supplementary Table S1 for details). This fact reflects the heterogeneity of the cohorts analyzed. Table 1 also shows a summary of the demographic and clinical information of the children involved. It should be noted that only polysomnographic recordings from the CHAT and CCSHS datasets included annotations of the time location of apneic and hypopneic events. Thus, PSGs from CHAT and CCSHS were randomly and subject-wise allocated into 3 subsets to train ( $\approx$ 60%), validate ( $\approx$ 20%), and test ( $\approx$ 20%) the CNN model [23], [24], [25]. Regarding UofC and UofT, they could not be used to train the model as they did not contain annotations. Consequently, these were distributed into 2 subsets, validation ( $\approx$ 60%) and test ( $\approx$ 40%), to optimize the hyperparameters and evaluate the diagnostic performance of our CNN, respectively [22], [23], [25].

**TABLE 1:** Demographic and Clinical Information of the Children Involved in Our Study

	CHAT-Training	CCSHS-Training	CHAT-Validation	CCSHS-Validation	UofC-Validation	UofT-Validation	CHAT-Test	CCSHS-Test	UofC-Test	UofT-Test
Subjects (n)	1006 (61.4%)	309 (60.0%)	326 (19.9%)	103 (20.0%)	584 (60.0%)	327 (60.0%)	306 (18.7%)	103 (20.0%)	390 (40.0%)	218 (40.0%)
Age (years)	7.0 [2.0]	17.7 [0.5]	7.0 [2.0]	17.7 [0.6]	6.0 [5.0]	7.0 [7.4]	6.9 [2.0]	17.7 [0.5]	5.5 [6.0]	7.4 [8.2]
Males (n)	471 (46.8%)	153 (49.5%)	156 (47.9%)	49 (47.6%)	346 (59.2%)	177 (54.1%)	134 (43.8%)	58 (56.3%)	253 (64.9%)	116 (53.2%)
BMI (kg/m ${}^{{2}}$)	17.4 [6.1]	23.2 [6.0]	17.1 [6.4]	23.9 [6.2]	17.7 [6.6]	19.5 [12.1]	17.6 [6.0]	23.3 [7.0]	18.2 [5.9]	19.6 [12.1]
AHI (e/h)	2.6 [4.8]	0.8 [1.5]	2.4 [4.6]	0.9 [1.7]	4.1 [8.3]	2.4 [5.7]	2.3 [5.1]	0.8 [1.7]	3.3 [6.5]	2.3 [6.0]
No OSA (n)	219 (21.8%)	182 (58.9%)	69 (21.2%)	54 (52.4%)	96 (16.4%)	99 (30.3%)	67 (21.9%)	58 (56.3%)	75 (19.2%)	77 (35.3%)
Mild OSA (n)	496 (49.3%)	116 (37.5%)	168 (51.5%)	38 (36.9%)	229 (39.2%)	127 (38.8%)	148 (48.4%)	39 (37.9%)	169 (43.3%)	80 (36.7%)
Moderate OSA (n)	160 (15.9%)	7 (2.3%)	44 (13.5%)	6 (5.8%)	113 (19.4%)	51 (15.6%)	49 (16.0%)	3 (2.9%)	63 (16.2%)	28 (12.9%)
Severe OSA (n)	131 (13.0%)	4 (1.3%)	45 (13.8%)	5 (4.9%)	146 (25.0%)	50 (15.3%)	42 (13.7%)	3 (2.9%)	83 (21.3%)	33 (15.1%)

Data presented as median [interquartile range] or n (%). BMI = body mass index; AHI = apnea-hypopnea index; e/h = events/hour; OSA = obstructive sleep apnea; CHAT = Childhood Adenotonsillectomy Trial; CCSHS: Cleveland Children’s Sleep and Health Study; UofC = dataset from the University of Chicago; UofT = dataset from the University of Tennessee.

## III. Methods

**FIGURE 1.**
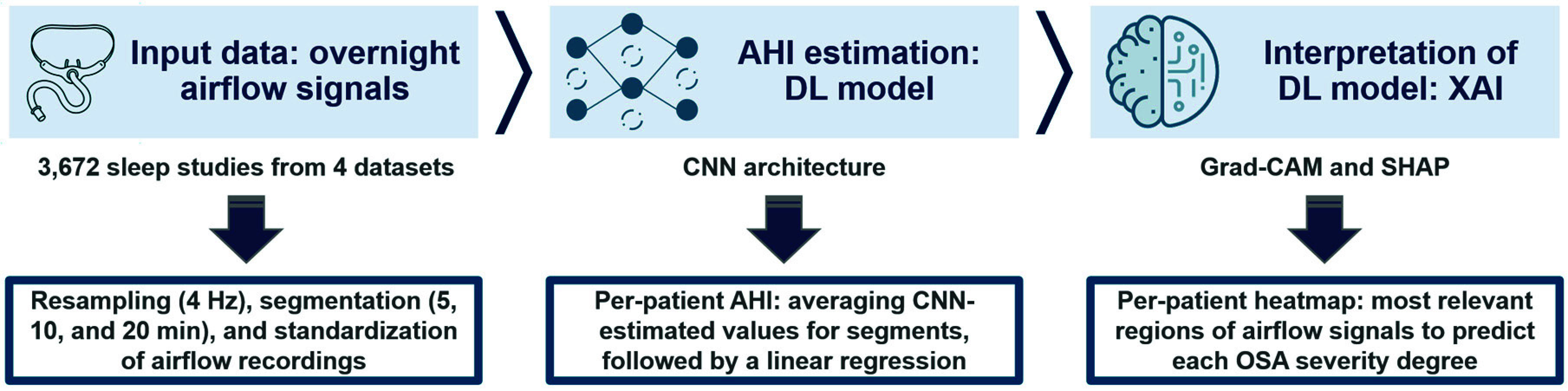
Workflow of the methodology proposed in our research study. AHI: apnea-hypopnea index; DL: deep-learning; CNN: convolutional neural network; XAI: explainable artificial intelligence; Grad-CAM: gradient-weighted class activation mapping; SHAP: shapley additive explanations; OSA: obstructive sleep apnea.

Fig. 1 presents the workflow of the methodology proposed in our research study. We designed, implemented, validated, and interpreted a CNN-based predictive model fed with overnight airflow information to aid in the pediatric OSA diagnosis. The following subsections describe the architecture of this model, as well as the metrics used to evaluate its diagnostic performance, and the methods applied to provide it with understandability.

### A. Signal Preprocessing

All airflow signals involved in our study were recorded using an oronasal thermistor sensor integrated into the polysomnographic equipment used during PSG in the different hospitals. The respiratory data, originally acquired at sampling frequencies from 20 to 512 Hz were down sampled to a common sampling rate of 4 Hz to homogenize them, as well as to reduce computational costs [26]. Since apneas and hypopneas can appear in clusters, the resampled signals were further segmented into 5-min, 10-min, and 20-min intervals, resulting in airflow segments with dimensions of 1,200, 2,400, and 4,800 samples, respectively. Subsequently, a z-score standardization process was applied to segments to normalize inter- and intra-subject baseline airflow levels [35]. As artifact-free signals might overfit the model and reduce its ability to handle and generalize noisy data in real cases, none of the airflow recordings underwent filtering or artifact removal processes [26]. This was a methodological choice, as training only with clean signals may bias the model toward idealized conditions. Preserving intrinsic variability and artifacts was intended to expose the CNN to realistic noise sources (e.g., movement or disconnections) and to improve its robustness in real-world clinical settings. Thereby, our CNN could discern between spurious and relevant airflow information, enhancing its ability to recognize and work with real pediatric respiratory data.

### B. CNN Model Architecture

**FIGURE 2.**
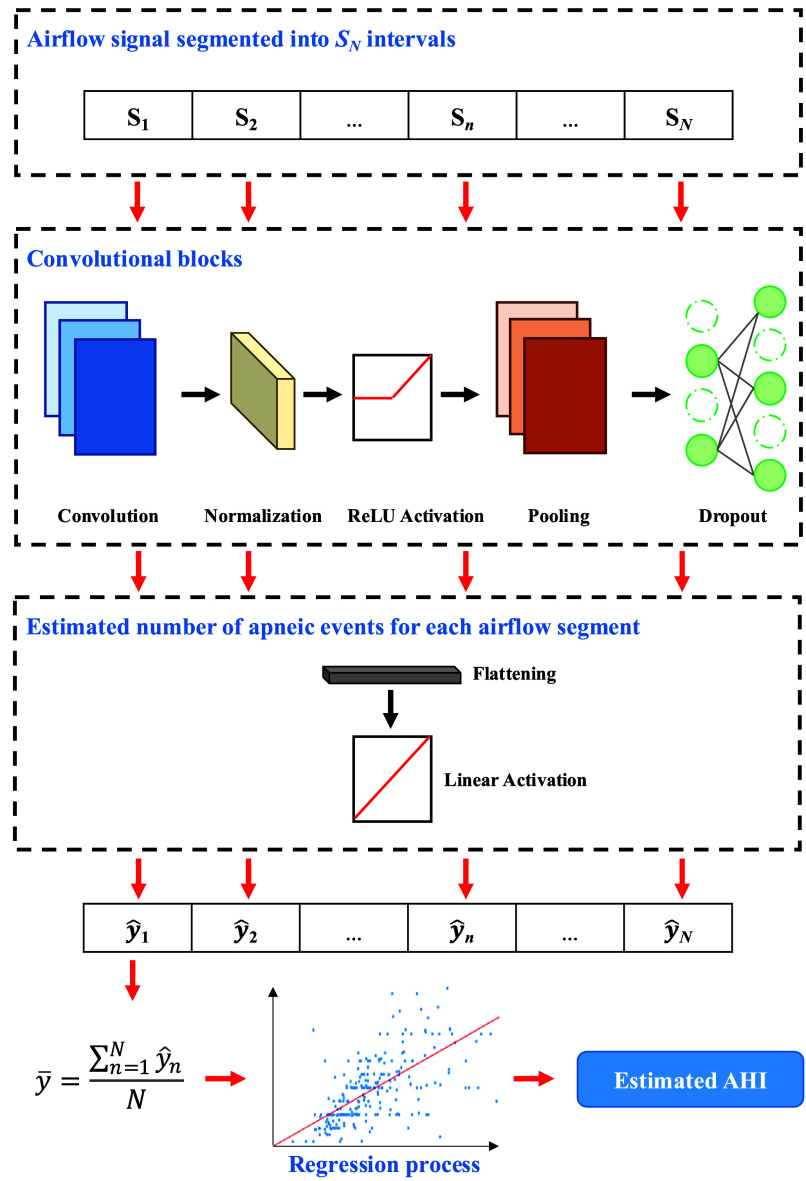
Outline of the convolutional neural network (CNN) architecture applied in study for predicting the obstructive sleep apnea severity in children from airflow signal. *N*: number of equal-length segments into which airflow signal is divided; S_1_, S_2_, …, $\text{S}_{N}$: segments of airflow signal; ReLU: Rectified Linear Unit; $\hat{y_{1}}$, $\hat{y_{2}}$, …, $\hat{y}_{N}$: number of apneic events estimated by the CNN per each signal segment $\text{S}_{N}$; $\bar{y}$: average of the predicted values $\hat{y_{N;}}$ AHI: apnea-hypopnea index.

A CNN architecture was chosen since it can automatically learn relevant features of physiological signals at different time and frequency scales, allowing the identification of complex patterns, such as spikes, waves, or specific events in time [20]. Moreover, convolutional layers can capture features that remain invariant to small temporal variations [20], [21], which is essential for airflow since OSA-associated patterns may fluctuate in time and amplitude. Accordingly, a visual scheme of architecture is presented in Fig. 2. The CNN receives as input the standardized segments of airflow signal (1,200; 2,400; or 4,800 samples). Subsequently, the data are processed through $N_{c}$ convolutional blocks, where $N_{c}$ —the number of convolutional blocks— is a tunable hyperparameter optimized during training. Each block consists of the following five consecutive layers [22], [23], [24]: 1D-convolution, normalization, activation, pooling, and dropout. In the 1D-convolution, 256 filters with a kernel size of $5\times 1$ and a stride of 1 are applied to the input data to extract the feature maps. The number of filters, kernel size, and stride remain constant across all convolutional blocks. This layer is followed by batch normalization and a non-linear activation process using the Rectified Linear Unit (ReLU) to identify the complex relationships [20], [21], [36]. Next, the dominant features of the map are obtained to reduce its dimensionality (max-pooling), and the rate of pooled activations ( $r_{drop}$) is randomly removed to decrease interdependence between neurons and thus minimize overfitting (dropout) [20], [21], [36]. After the $N_{c}$ convolutional blocks, a flattening layer reshapes the feature maps into a single 1D array. Finally, a linear activation layer performs the linear combination of its elements to estimate the number of apneic and hypopneic events contained in each airflow segment [22], [23]. Like previous studies [22], [23], [35], the AHI of each pediatric subject was obtained as the average of the CNN-estimated values for all segments contained in their airflow signal, followed by a linear regression process to correct possible overestimations related to discrepancies between total sleep time and total recording time.

To obtain the optimal configuration for the segment size and the hyperparameters $N_{c}$ (explored values for the number of convolutional blocks: 6, 7, and 8) and $r_{drop}$ (search space for the dropout rate: 0.0, 0.1, and 0.2), we used the following configuration: adaptive moment estimation algorithm (Adam optimizer) with an initial learning rate of 0.001 to optimize network weights [37]; batch sizes of 64 (20-min segments), 128 (10-min segments), and 256 (5-min segments) with a random data shuffling strategy; a learning rate decay by a factor of 2 after 10 epochs of no reduction in the loss of the validation set; and early stopping after 30 epochs of no improvement, being the best model selected as those with the lowest validation loss. According to other studies from our research group [23], [25], [35], we utilized the Huber loss function with a delta parameter set to 1 due to its suitability in handling random distributions [38]. The proposed CNN model was developed in Python 3.8.13 through the TensorFlow 2.8.0 library. The training and optimization processes took place on a high-performance computer equipped with 64 GB of RAM, Intel Core i9-11900KF 3.50 GHz processor, and NVIDIA GeForce RTX 3080 Ti graphic card.

### C. CNN Model Performance

In order to assess the agreement between the AHI estimated by our CNN and the actual values obtained from PSG, we utilized the intraclass correlation coefficient (ICC) and the root mean-squared error (RMSE). Additionally, Bland-Altman plots were used for visual analysis of this agreement [39]. In all datasets involved, the overall diagnostic performance of our predictive model was globally evaluated by means of Cohen’s kappa (*k*) [40], confusion matrix, and accuracy (Acc_4_) for 4 classes. The performance for OSA severity degrees (AHI cutoffs 1, 5, and 10 e/h) was assessed using a binary classification approach (i.e., AHI $< $ 1 e/h vs. AHI $\ge$ 1 e/h, AHI $< $ 5 e/h vs. AHI $\ge$ 5 e/h, and AHI $< $ 10 e/h vs. AHI $\ge$ 10 e/h) through common statistical metrics: sensitivity (Se), specificity (Sp), accuracy (Acc), positive and negative predictive values (PPV and NPV, respectively), and positive and negative likelihood ratios (LR+ and LR-, respectively).

### D. CNN Model Interpretability

Giving interpretability to a DL model involves understanding and explaining the underlying reasons that motivate the model to make its prediction [27], [29]. Although CNNs are powerful for processing complex data, their internal workings can be opaque due to their high complexity and number of parameters [27], [29], [30]. To deal with this drawback and improve the reliability of predictive models, there is a growing and necessary use of XAI techniques [27], [28], [29]. Particularly, we applied Grad-CAM and SHAP due to the fact that they are the most widely used XAI techniques and have been shown to provide transparency and interpretability to CNN networks [27], [28], [30].

Grad-CAM is a *post-hoc* technique used in the field of DL to visualize and understand which parts of an image or signal are important for decision-making and predictions [27]. Based on the gradients from the convolutional layer output, this technique generates localization maps that highlight the relevant and influential regions for the CNN-derived estimates. Thereby, the input goes through the neural network to the target convolutional layer. Once this has been fed forward and the output of the convolutional layer has been obtained, the gradients of the model output with respect to the feature map presented in each convolutional layer are calculated using backpropagation [27], [41]. These gradients are then globally averaged, weighing each feature map by the importance of the gradient. Finally, the information from the weighted gradients for each feature map is combined using aggregation operations (usually, a weighted average), followed by a ReLU activation process to distinguish the features that have a positive influence on the model prediction and thus achieve better localization. In order to highlight both simple and complex OSA-associated patterns in airflow signal, a heatmap was obtained for each convolutional layer of the $N_{C}$ blocks by applying Grad-CAM [25]. Subsequently, all intermediate heatmaps were normalized, resized, and averaged to derive the final heatmap [25], which highlights with higher values (warmer colors) the regions of the signal that contribute to the final prediction of the CNN the most [27], [41]. As pediatric OSA introduces amplitude, phase, and frequency variations in airflow signals, higher heatmap values are expected when the CNN estimates the presence of apneic and hypopneic events.

Regarding SHAP, it is a *post-hoc* explainability technique based on Shapley values from cooperative game theory, which assigns each player a value that represents their contribution to the game [28]. In the context of model interpretability, each feature is considered a player that contributes to the final prediction of that model. Therefore, the SHAP value for a particular input feature in a specific prediction is the expected contribution of that feature to the difference between the prediction and the expected value of the model [28]. This value captures how the inclusion or exclusion of a feature influences the model’s prediction. Accurately calculating Shapley values involves evaluating all possible combinations of features to determine the contributions of each of them. However, this is computationally expensive and not feasible for large datasets or complex models. Consequently, approximate approaches are applied [28], [42]. In our study, we use the Deep-SHAP, a specially designed technique for neural networks that combines the DL Important FeaTures (Deep-LIFT) and SHAP approaches [28], [42]. Assuming the linearity of the model and the independence of the input features, Deep-LIFT estimates the contribution of each of them by applying backpropagation and linear composition rules [43]. Lundberg and Lee demonstrated that this approach can be used to approximate SHAP values, resulting in Deep-SHAP [28]. The underlying idea of this technique is to calculate SHAP values of simple neural network components to obtain attributions more quickly and accurately for the whole model. The compositional approximation derived from Deep-SHAP results from summing the difference between the expected model output in the previous simple components and the current output of the model [28], [42]. Thereby, high Shapley values in absolute magnitude indicate that the feature is relevant for the model-derived prediction. In the OSA context, higher SHAP positive values (positive contributions: red regions) are expected when the CNN estimates apneas and hypopneas in the airflow segments. In contrast, high negative values (negative contributions: blue regions) are expected in respiratory patterns that are not OSA-informative and that lead to errors in prediction.

## IV. Results

### A. Optimal Configuration of the CNN Model

A hyperparameter optimization process was conducted for our CNN model. Different combinations were explored to determine the optimal segment size, testing intervals of 5, 10, and 20 min of airflow signal. We also varied the number of convolutional blocks ( $N_{c}$) that constitute the model, experimenting with 6, 7, and 8 convolutional blocks. Additionally, 0.0, 0.1, and 0.2 were considered as possible optimal values for $r_{drop}$. Thereby, CNN was trained with the corresponding training subset (1,006 airflow signals from CHAT and 309 from CCSHS). Subsequently, each model configuration —defined by a specific combination of segment size, $N_{c}$, and $r_{drop}$ value— was evaluated in the validation subset (326 airflow signals from CHAT, 103 from CCSHS, 584 from UofC, and 327 from UofT). The continuous AHI values predicted by the CNN were used to evaluate diagnostic performance by establishing OSA severity groups (no OSA, mild, moderate, and severe OSA). Cohen’s kappa was then used in the validation subset as a metric to assess the agreement between the model-derived severity and that assigned by the sleep medicine expert manually from PSG recordings. Among all the configurations assessed (see Table S2 included in Supplemental Items), the combination that achieved the highest *k* involved 10-min segments, 7 convolutional blocks, and a $r_{drop}$ of 0.1. This particular configuration exhibited greater agreement in the validation dataset, with a $k =$ 0.3688.

### B. CNN Model Performance in Test Subsets

**FIGURE 3.**
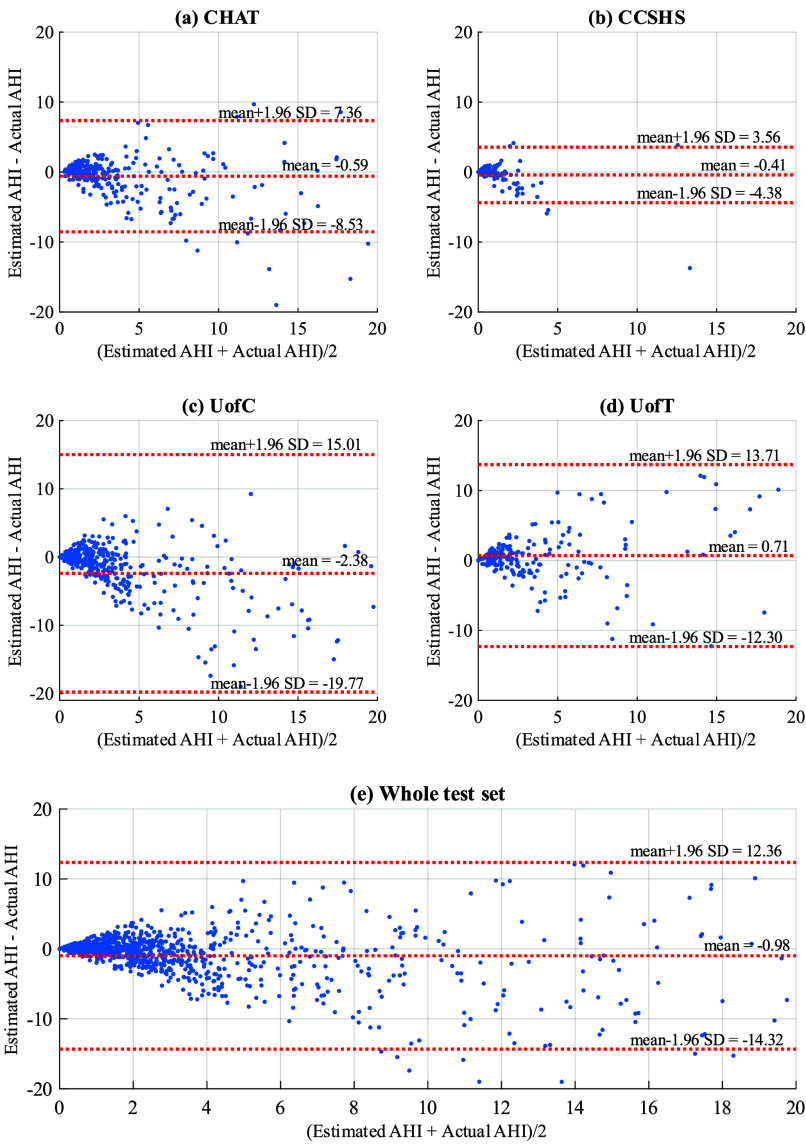
Bland-Altman plots obtained from the apnea–hypopnea index (AHI) estimated by the convolutional neural network (CNN) model and the one derived from polysomnography in the test sets of (a) Childhood Adenotonsillectomy Trial — CHAT, (b) Cleveland Children’s Sleep and Health Study — CCSHS, (c) University of Chicago — UofC, (d) University of Tennessee — UofT, and (e) the whole test set. The plots also show the mean difference and the limits of agreement, calculated as the mean ± 1.96 × standard deviation (SD).

Across all four datasets, the test set results revealed a significant positive concordance between CNN-estimated AHI and PSG-derived AHI, with ICC values of 0.87 for CHAT, 0.76 for CCSHS, 0.69 for UofC, and 0.83 for UofT (ICC = 0.77 for the whole test set). The obtained RMSE was 4.09 for CHAT, 2.06 for CCSHS, 9.17 for UofC, and 6.66 for UofT, with an overall RMSE of 6.87 for the whole test set. These results are consistent with the Bland-Altman plots in Fig. 3, which show a high level of agreement between both variables. Most data points fell within the goodness-of-fit limits, and no apparent bias was present (mean difference ranging from -2.38 to 0.71), further validating the model’s reliability across different AHI ranges. Therefore, our model not only provides statistical concordance but also maintains clinical consistency in its estimates.

**FIGURE 4.**
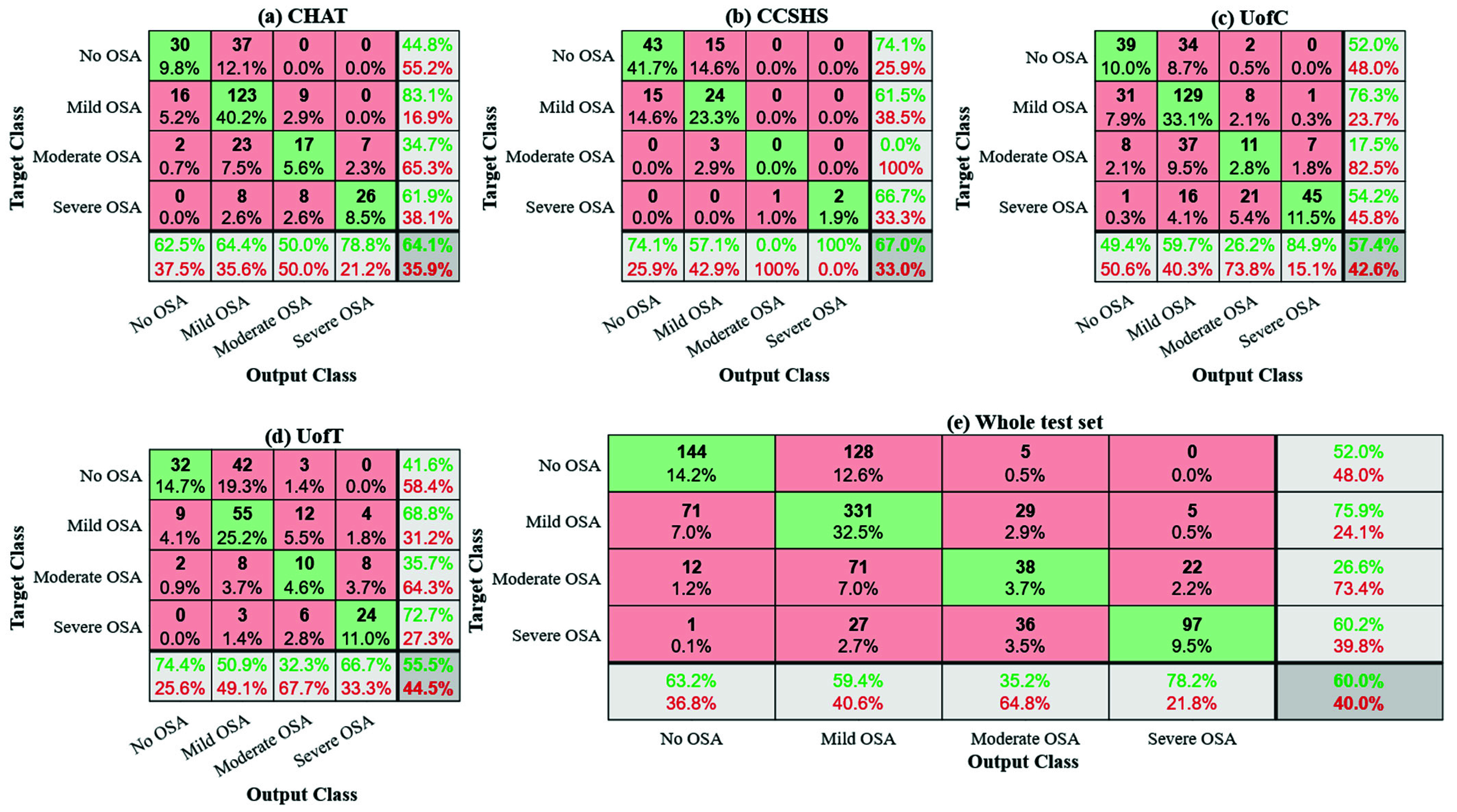
Confusion matrices obtained by our convolutional neural network (CNN) model in the test sets of (a) Childhood Adenotonsillectomy Trial — CHAT, (b) Cleveland Children’s Sleep and Health Study — CCSHS, (c) University of Chicago — UofC, (d) University of Tennessee — UofT, and (e) the whole test set. Obstructive sleep apnea (OSA) severity degrees: no OSA (1 event/h (e/h) $< $ apnea-hypopnea index (AHI)), mild (1 e/h $\le$ AHI $< $ 5 e/h), moderate (5 e/h $\le$ AHI $< $ 10 e/h), and severe OSA (AHI $\ge$ 10 e/h).

Fig. 4 shows the confusion matrices generated by the CNN model for the test set of each dataset. From these matrices, the overall diagnostic performance of the model, as well as the model performance at the conventional clinical severity thresholds (1, 5, and 10 e/h) were derived. It is worth noting that the model achieved a 4-class accuracy between 55.50% and 66.99% across the different datasets, along with a Cohen’s k ranging from 0.37 to 0.43 (Table 2). Similarly, the CNN model achieved high diagnostic accuracy across all thresholds (Table 3), particularly at 5 and 10 e/h (Acc values for binary classification ranging from 81.28% to 97.09% and from 88.21% to 99.03%, respectively). Additionally, the model demonstrated high LR+ at the 10 e/h threshold, reaching values as high as 11.21 or greater. These metrics highlight the robust performance of the model in distinguishing between different severities of sleep apnea, with particularly strong results in the more clinically relevant thresholds (5 and 10 e/h).

**TABLE 2:** Global Performance of Our Convolutional Neural Network (CNN) Model in the Test Sets of CHAT, CCSHS, UofC, and UofT Datasets, and the Whole Test Set

	k	Acc ${}_{{4}}$(%)	ICC	RMSE
CHAT	0.43	64.05	0.87	4.09
CCSHS	0.37	66.99	0.76	2.06
UofC	0.37	57.44	0.69	9.17
UofT	0.37	55.50	0.83	6.66
Overall	0.40	59.98	0.77	6.87

$k =$ Cohen’s kappa for 4 classes; $\text{Acc}_{4} =$ accuracy for 4 classes; ICC = intraclass correlation coefficient; RMSE = root mean-squared error; CHAT = Childhood Adenotonsillectomy Trial; CCSHS: Cleveland Children’s Sleep and Health Study; UofC = dataset from the University of Chicago; UofT = dataset from the University of Tennessee.

**TABLE 3:** Diagnostic Performance of Our Convolutional Neural Network (CNN) Model for the AHI Cutoffs 1, 5, and 10 Events/Hour (e/h) Using a Binary Classification Approach in the Test Sets of CHAT (306 Subj.), CCSHS (103 Subj.), UofC (390 Subj.), and UofT (218 Subj.) Datasets, as Well as in the Whole Test Set (1,017 Subj)

cutoff	Test set	Se (%)	Sp (%)	Acc (%)	PPV (%)	NPV (%)	LR+	LR-
1 e/h	CHAT	92.47	44.78	82.03	85.66	62.50	1.67	0.17
	CCSHS	66.67	74.14	70.87	66.67	74.14	2.58	0.45
	UofC	87.30	52.00	80.51	88.42	49.37	1.82	0.24
	UofT	92.20	41.56	74.31	74.29	74.42	1.58	0.19
	Overall	88.65	51.99	78.66	83.14	63.16	1.85	0.22
5 e/h	CHAT	63.74	95.81	86.27	86.57	86.19	15.23	0.38
	CCSHS	50.00	100.00	97.09	100.00	97.00	Inf.	0.50
	UofC	57.53	95.49	81.28	88.42	78.98	12.76	0.44
	UofT	78.69	87.90	85.32	71.64	91.39	6.50	0.24
	Overall	63.49	94.53	85.25	83.19	85.86	11.61	0.39
10 e/h	CHAT	61.90	97.35	92.48	78.79	94.14	23.35	0.39
	CCSHS	66.67	100.00	99.03	100.00	99.01	Inf.	0.33
	UofC	54.22	97.39	88.21	84.91	88.72	20.81	0.47
	UofT	72.73	93.51	90.37	66.67	95.05	11.21	0.29
	Overall	60.25	96.85	91.05	78.23	92.83	19.10	0.41

AHI = apnea-hypopnea index; e/h = events/hour; Se = sensitivity; Sp = specificity; Acc = accuracy; PPV = positive predictive value; NPV = negative predictive value; LR $+ =$ positive likelihood ratio; LR- = negative likelihood ratio; CHAT = Childhood Adenotonsillectomy Trial; CCSHS: Cleveland Children’s Sleep and Health Study; UofC = dataset from the University of Chicago; UofT = dataset from the University of Tennessee.

### C. CNN Model Interpretability in Test Subsets

**FIGURE 5.**
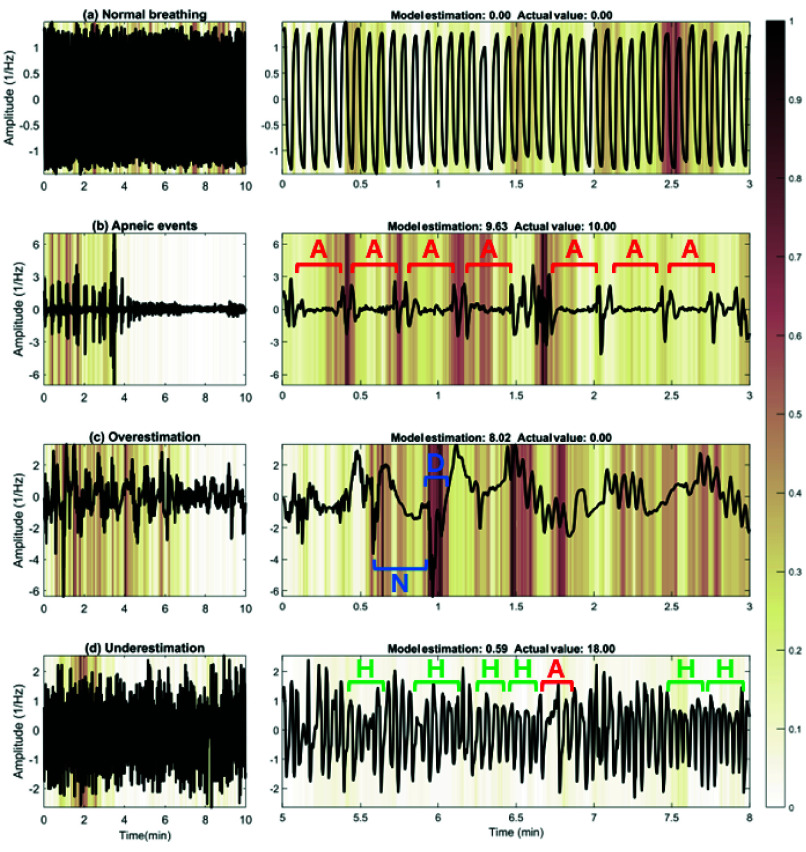
Gradient-weighted class activation mapping (Grad-CAM) heatmaps generated from 10-min segments of (a) normal breathing, (b) apneic events [A], (c) overestimation with noise artifact [N] and oxygen desaturation [D] events, and (d) underestimation with hypopneic [H] and apneic events [A] scored by specialist physicians in the Childhood Adenotonsillectomy Trial (CHAT) dataset.

**FIGURE 6.**
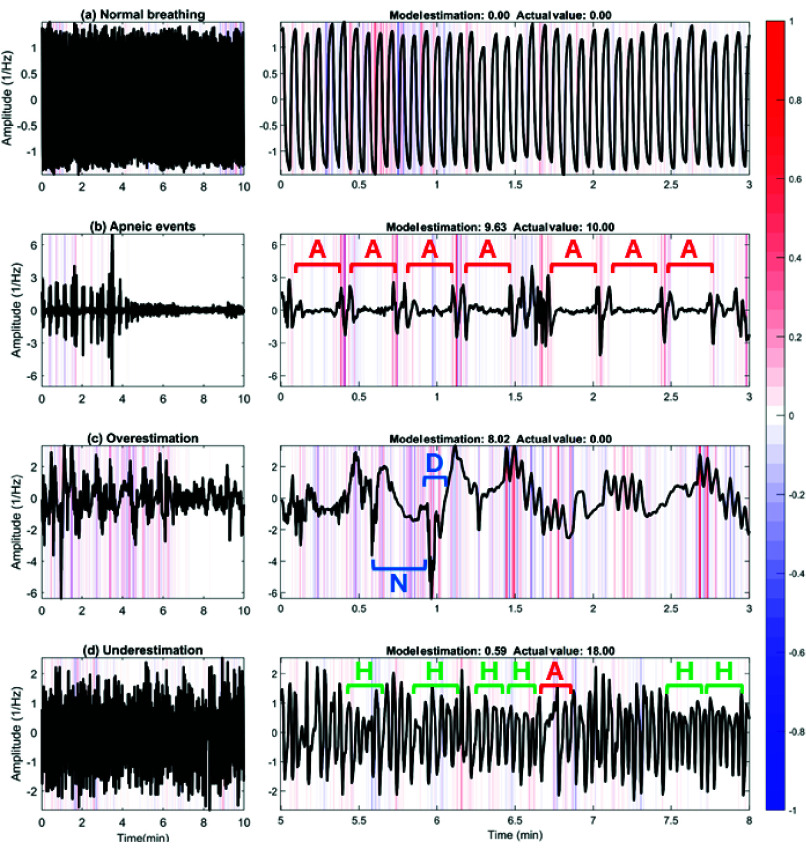
Shapley additive explanation (SHAP) plots generated from 10-min segments of (a) normal breathing, (b) apneic events [A], (c) overestimation with noise artifact [N] and oxygen desaturation [D] events, and (d) underestimation with hypopneic [H] and apneic events scored by specialist physicians in the Childhood Adenotonsillectomy Trial (CHAT) dataset.

Fig. 5 and 6 present four examples of the results obtained with Grad-CAM and SHAP, respectively, illustrating how the CNN model performed in different scenarios at the CHAT dataset. In cases where the predictions of the model were correct, we observe (a) segments of normal breathing and (b) apneic events. For the latter, the model primarily focused on the onset and offset of the apneic event to make its prediction. In contrast, the model paid almost no attention to the regions of normal breathing, where the signal hardly changes. These figures also highlight cases where the predictions of the model were incorrect, particularly in (c) overestimation and (d) underestimation of the number of apneic events. Comparing both methods, we observed that Grad-CAM tended to highlight specific transition regions in airflow, while SHAP distributed importance more broadly. This led Grad-CAM to be more effective at identifying abrupt changes in airflow amplitude, while SHAP was useful for interpreting how minor variations and irregularities influence the predictions of the model. Fig. S1 and S2 included as Supplemental Items provided additional results of Grad-CAM and SHAP for the CCSHS dataset, further supporting these findings.

## V. Discussion

In this study, we designed, implemented, and validated a CNN model for automatic detection of respiratory patterns associated with pediatric OSA. We incorporated explainability and transparency into our model by means of XAI techniques, allowing healthcare professionals to understand how the model reaches its decisions and to enable trust in the diagnostic accuracy of the model. The model demonstrated promising performance in identifying OSA and achieved high accuracy for severe and mild cases, although moderate OSA posed the greatest classification challenge. Moreover, it became apparent that the model prioritized regions where airflow amplitude suddenly changes.

### A. CNN Model Optimization

The optimization results revealed a clear interaction among segment size, the number of convolutional blocks, and the dropout rate in the AHI prediction. Higher *k* values indicated a better agreement between the actual and predicted AHI, particularly with segment sizes of 10 min and $N_{C}$ of 7 ( $k =$ 0.3688). This configuration would not only enhance the identification of the specific features of airflow, but also the predictive accuracy of our model. Although a shorter segment generates a larger quantity of data, 5-min segments may not capture all the relevant information related to OSA. As in many times apneic events appear in clusters (Fig. 5-6 (b) and (d)), using 5-min segments may hinder the ability of the CNN to learn respiratory patterns that require a broader temporal context, resulting in lower *k* values. Configurations with a segment duration of 20 minutes also tended to show reduced performance. In contrast, the results obtained suggest that 10-min segments provide sufficient temporal context to capture complex respiratory patterns, thereby enhancing the ability of our model to make accurate predictions.

Regarding $N_{C}$, increasing the number of blocks to 7 improved the ability of the CNN to extract relevant features from airflow. This increase in model complexity allows for the identification of more subtle respiratory patterns, which are essential for accurate apnea and hypopnea detection. An increase to 8 convolutional blocks showed improvements in performance with 5 and 20-min segments, highlighting that there is a trade-off between segment size and the number of blocks used in the model. Interestingly, by utilizing 8 convolutional blocks did not lead to significant improvements in the performance with 10-min segments, suggesting that additional complexity does not always result in better predictive performance. Furthermore, the tested $r_{drop}$ did not consistently enhance the performance of our model,

indicating that while regularization is essential, its impact varies depending on other architectural parameters. These findings highlight the importance of optimizing segment size, model architecture, and convolutional hyperparameters to improve model performance in estimating AHI in pediatric population. They suggest that a strategic combination of these elements may result in greater effectiveness in predicting complex respiratory conditions.

### B. CNN Model Performance

Our CNN model demonstrated a strong correlation between the estimated and actual AHI values (ICC = 0.77 and RMSE = 6.87 in the overall test set), with a slight overall underestimation bias (mean bias $= -0.98$ in Fig. 3 (e)). The differences between the estimated and actual AHI values were mostly within the margins of agreement (975/1016 estimations). Notably, these differences were centered around zero, indicating that the model generally provided accurate predictions, though with slight variations, particularly for higher AHI values. The CHAT dataset yielded the highest agreement (ICC = 0.87, RMSE = 4.09, mean bias $= -0.59$, with 289/306 estimations within the limits of agreement), followed by CCSHS (ICC = 0.76, RMSE = 2.06, mean bias $= -0.41$, 96/102 estimations within range), both showing narrow limits of agreement and low dispersion. This improved performance on the CHAT dataset could be partially attributed to the fact that most of the recordings used during training originated from this dataset, allowing the model to better learn its specific signal characteristics. The lower variability in CCSHS could be attributed to its lower AHI values compared to CHAT. Performance declined in the UofC dataset (ICC = 0.69, RMSE = 9.17, mean bias = -2.38, 375/390 estimations within range), tending to underestimate the actual AHI more than in the previous datasets. This was likely due to greater signal variability, differences in data acquisition devices, and/or variations in inclusion protocols, which also resulted in wider agreement limits (−19.77 to 15.01). Similarly, while UofT showed a high correlation (ICC = 0.83) and a slight bias toward overestimating the AHI (mean bias = 0.71, RMSE = 6.66, 206/218 estimations within range), its broader limits of agreement suggested greater variability in some predictions. These significant differences in agreement limits highlighted the influence of the AHI amplitude ranges contained in each dataset.

Regarding classification accuracy, the model effectively identified children without OSA and those with mild OSA, achieving high sensitivity in both CHAT and CCSHS. While some misclassifications occurred in cases of moderate-to-severe OSA, the high accuracy of our CNN at 5 and 10 e/h reinforces its effectiveness in identifying these patients. Moreover, the positive likelihood ratio (LR+) values $\ge$ 10 at these thresholds suggest that the model is a robust indicator for detecting the most critical stages of the disease—an essential aspect in clinical contexts. This tendency was consistent across most of the analyzed datasets, supporting the potential of the retained CNN model as a reliable tool for both initial pediatric OSA screening and the early detection of severe cases. Similar performance was observed in UofC and UofT, with a particularly high diagnostic accuracy for severe OSA. Notably, most classification errors occurred between adjacent categories, reflecting the inherent challenge of distinguishing between different pediatric OSA severity levels. In this regard, the misclassified participants had an actual AHI close to threshold values, with 48.39% within 1 ± 0.5 e/h, 11.33% within 5 ± 0.5 e/h, and 9.89% within 10 ± 0.5 e/h, which potentially suggests ambiguity in borderline cases. Despite this, the model achieved four-class accuracies as high as 66.99% and *k* values of up to 0.43, highlighting its effectiveness in successfully classifying patients into the different OSA severity groups. Thus, the following screening protocol can be derived from the obtained results: i) if our CNN estimates an AHI $< $ 1 e/h, rule out the presence of OSA as these children most likely (95.83% in CHAT, 100% in CCSHS, 88.61% in UofC, and 95.35% in UofT) have an actual AHI $< $ 5 e/h. If symptoms persist, referral to PSG is recommended; ii) if the model predicts 1 e/h $\le$ AHI $< $ 5 e/h, recommend undergoing PSG since there are uncertainties concerning the actual diagnosis of the child; iii) if the CNN-derived diagnosis is 5 e/h $\le$ AHI, recommend treatment as these children most likely (100% in CHAT, 100% in CCSHS, 97.89% in UofC, and 95.52% in UofT) have at least mild OSA. Moreover, patients with a predicted AHI $\ge$ 10 should undergo additional follow-up, as they are likely to experience persistent OSA after treatment.

By following the proposed screening protocol would lead to a 45.23% reduction in the number of full PSGs (37.58% in CHAT, 59.22% in CCSHS, 44.62% in UofC, and 50.46% in UofT). Such a reduction of PSGs not only would optimize healthcare resources but also alleviate the burden on both patients and clinicians. Reducing PSG use with this screening protocol could improve patient experience and simplify pediatric OSA diagnosis, particularly at 10 e/h, where the CNN performed best. Since untreated OSA can cause serious health complications, such as cardiovascular and cognitive issues [7], [8], [44], early and accurate detection is essential for timely intervention. Identifying high-risk children, i.e., those with more severe disease through our screening protocol would allow for timely and tailored treatments, which often require invasive procedures such as tonsillectomy [7], [8], [44]. Consequently, our CNN could enable timely and targeted treatment and reduce long-term health risks.

### C. CNN Model Interpretability

The interpretability analysis with Grad-CAM and SHAP revealed that the model processes segments with normal breathing and apneic events differently. In the normal breathing segment, the CNN paid almost no attention to airflow signal characteristics when making its prediction. This was an expected finding given that in this region there were no relevant alterations caused by apnea or hypopnea events. However, both Grad-CAM and SHAP revealed that the model focused on particular regions of airflow signal during periods of normal breathing (e.g., a dark warm color displayed in the Grad-CAM heatmap of Fig. 5 (a) at minute 2.5, or in the SHAP plot of Fig. 6 (a) at minute 0.6). This observation suggests that the model may not only be interpreting the typical signal amplitude changes associated with OSA, but also considering other physiological and intrinsic properties of airflow, such as the respiratory rate or the distance between respiratory cycles. In segments containing apneas (Fig. 5-6 (b)), both Grad-CAM and SHAP successfully pointed out these regions, showing the strong focus of the model on the beginning and end of each apneic event. Thus, SHAP confirmed that these transition points contributed positively to the predictions of CNN. This behavior aligns with the physiological changes experienced by children with OSA, since the beginning and end of an apnea are often characterized by abrupt changes in airflow signal due to the interruption and resumption of respiratory flow. Consequently, this suggests that the CNN accurately identifies the critical points of airflow disruption and recovery, which are key indicators of OSA. In contrast, the model stopped focusing on airflow signal characteristics when normal breathing resumed (after min 4 in Fig. 5-6 (b)), demonstrating its ability to distinguish between normal and pathological breathing patterns. According to the findings derived from the diagnostic performance analysis in the previous section, this selective attention of our CNN would have relevant clinical implications as it reduces the risk of false positives, efficiently captures significant airflow changes associated with the pathophysiology of apnea, and enhances diagnostic accuracy. Thus, our CNN model could be a powerful tool to automatically identify the OSA-related respiratory patterns and contribute to their interpretation.

The application of XAI techniques not only enabled the correct identification of favorable cases but also pointed out instances where the model made mistakes. These errors were particularly significant as they provided a valuable opportunity to understand how the model makes its decisions and how it could be improved. In a particular case of overestimation, our CNN predicted 8.02 apneic events, while the actual estimation was 0.00 events (Fig. 5–6 (c)). A detailed analysis of this airflow segment revealed several factors that could have contributed to the overestimation. Firstly, we observed that sudden changes in the airflow amplitude can be caused not only by apneic events but also by physiological disturbances, such as the movements of the child during PSG recording. According to the recording annotations, medical specialists marked artifacts at min 0.7 and 6.1 of this segment. It is possible that the model interpreted these alterations in the signal as apneic events, leading to overestimation. Additionally, several oxygen desaturations not associated with apneic or hypopneic events occurred from min 0.9 to 9.8. These desaturations, observed in the recording annotations, could be linked to other pathologies distinct from OSA, such as asthma or bronchiolitis, which affect the breathing pattern without generating apneas. Such conditions may disrupt the natural functioning of the respiratory system and, consequently, the airflow signal. It is possible that this fact led CNN to misinterpret these changes as signs of apnea, contributing to the overestimation. Further similar artifact-induced behaviors of the model can be found in Supplementary Fig. S1(c) and S2(c), which illustrate cases where noise and arousals not associated with respiratory events affected the airflow signal and led to mispredictions. In a particular case of underestimation (Fig. 5–6 (d)), the model predicted only 0.59 events, while the actual estimation was 18.00 events. Upon comprehensively reviewing this segment, we found two clear apneic events at min 6.7 and 8.1, along with 16 hypopneas marked by specialists in the recording annotations. However, the reductions in airflow signal amplitude associated with these events were not as pronounced or abrupt, suggesting that CNN might be failing to detect subtle disruptions in airflow. This indicates a potential limitation of our model in identifying less evident hypopneas, which cause smaller changes in airflow signal characteristics than complete breathing cessations.

When comparing the XAI methods, we observed that Grad-CAM and SHAP provided complementary approaches for interpreting CNNs predictions in pediatric OSA detection, supporting their successful application in this context [25]. Both succeeded in highlighting apneic events and other significant respiratory patterns in airflow, although they did so in different ways. Grad-CAM was particularly effective at emphasizing the more pronounced transitions in the signal, such as the onset and offset of apneas, making it suitable for identifying prominent respiratory events where the signal experiences abrupt amplitude changes. While its coarser resolution provided clearer visualizations and reduced background noise, it also led to the occasional oversight of less pronounced events (e.g., Fig. 5 (b) at min 2.1 and 2.5). On the other hand, SHAP distributed relevance more broadly and offered a finer resolution. This allowed it to highlight respiratory patterns characterized by small changes in airflow that could have been relevant in the CNN’s decision-making process. This ability to capture more subtle variations was crucial in cases where respiratory events might not be as pronounced or evident (e.g., the onset and offset of apnea events presented at min 2.1 and 2.5 in Fig. 6 (b)). However, due to this broader and more global approach, SHAP also highlighted areas of airflow where no relevant OSA-related events were present, which introduced background noise into the visualization and may have made it difficult to distinguish between true apneic events and artifacts or noise. It is also worth noting that Grad-CAM was computationally less expensive and more efficient in terms of required resources, making it a suitable choice for real-time use. In contrast, SHAP had a considerably higher computational cost, which could be a limitation in automatic diagnostic applications requiring real-time processing or resource-limited devices. Despite this, SHAP is especially useful when a more precise understanding of the signal is required. With XAI methods respective advantages and disadvantages, both were complementary for interpreting our CNN model. Grad-CAM was effective in identifying abrupt transitions and reducing visual noise, while SHAP provided a detailed view by capturing events with less evident changes that could have been missed in Grad-CAM. Thus, the use of XAI significantly improves the interpretation of airflow-based CNN; Grad-CAM can be used to filter pronounced apneic events and SHAP to provide deeper insights into subtle patterns, which would be key to accurately identifying respiratory patterns associated with pediatric OSA.

### D. Clinical Applicability of the Explainable CNN Model

Our explainable CNN model for pediatric OSA diagnosis offers numerous clinical advantages, particularly in optimizing diagnosis and management across various settings. The model enhances speed and objectivity in clinical decision-making by automating the diagnostic process and delivering early results that help physicians make faster and accurate clinical decisions. This reduces the waiting time for treatment and improves patient outcomes. Additionally, integrating techniques like Grad-CAM and SHAP boosts the interpretability of CNN. This allows clinicians to gain clear, objective insights into apneic events and OSA severity, which in turn supports more accurate treatment decisions, ranging from lifestyle changes to surgery.

Using only a single-channel airflow, the simplicity of our model makes it ideal for use in primary care clinics for initial OSA assessments. It does not require complex equipment like PSG, allowing general physicians to identify children who need further studies or interventions, thus streamlining the diagnostic process and reducing unnecessary specialist referrals. In sleep clinics, the model can support PSG by preselecting or rapidly evaluating patients, and optimizing resources by reserving PSG for more complex cases. It is also advantageous in areas with limited medical resources, as it can be used by primary care physicians without advanced hospital infrastructure, reducing disparities in access to healthcare.

The model can also be applied to home settings, providing comfort for patients and offering more accurate sleep pattern assessments. This is particularly beneficial for pediatric patients, who often struggle with in-lab PSG procedures. Our CNN is ideal for mass screening of high-risk populations, including children with obesity or craniofacial anomalies [3], [4], and can be used in school or pediatric clinical screening programs. Additionally, it allows for continuous monitoring of pediatric patients already undergoing treatment, enabling rapid adjustments to interventions and improving disease management without overburdening hospital resources.

In conclusion, the clinical applications of our explainable CNN model are broad and groundbreaking. From primary care screening and home diagnosis to continuous monitoring and follow-up, the model offers an accessible, cost-effective, and efficient way to diagnose and manage pediatric OSA. Thus, this approach would improve clinical outcomes and quality of life by reducing the reliance on complex and costly PSGs. Beyond these potential applications, our results also highlight a concrete step toward the clinical translation of explainable DL models into pediatric practice, supporting early diagnosis strategies and improving resource allocation in healthcare.

### E. Comparison With Previous State-of-the-Art Studies

When comparing the results of our model with other previous studies focused on pediatric OSA diagnosis from airflow signal, it was evident that our DL-based approach offered several significant advantages over traditional FE methods. As shown in Table 4, our model performed within the accuracy range for 1 e/h and achieved the highest diagnostic accuracy for 5 and 10 e/h in the UofC dataset—the one used in all compared state-of-the-art studies—as well as in the remaining datasets, outperforming other FE-based studies, such us recurrence or bispectral features [15], [16], [17], [18], [45]. Additionally, our CNN attained the highest specificity across all three thresholds. Although other research reported higher sensitivity, they were often associated with significant drops in specificity. In contrast, our model maintained a strong balance between sensitivity and specificity, which was crucial for conducting a more robust diagnosis. This performance highlighted the ability of the CNN to accurately differentiate between the various OSA severity degrees, minimizing overdiagnosis, and preventing the misclassification of patients without OSA or with mild degree as more severe cases. Furthermore, it underscored the effectiveness of DL methods, particularly CNNs, in improving the diagnostic ability of airflow signal by capturing complex patterns without the need for manual feature management, as required in traditional approaches.

**TABLE 4:** State-of-the-Art Studies Focused on the Pediatric OSA Diagnosis From Single-Channel Airflow Recordings

Study	Methods: Extraction / Selection / Classification / XAI / Validation	#Subjects (total/test)	Cutoff (e/h)	Se (%)	Sp (%)	Acc (%)
Barroso-García et al. 2017 [45]	Spectral and non-lineal features / FSLR / LR / - / K-fold & Holdout	501/251 UofC	1	60.5	58.6	60.0
			5	65.0	80.6	76.0
			10	83.3	79.0	80.0
Barroso-García et al. 2020 [15]	Recurrence features / FCBF / BY-MLP / - / Bootstrap & Holdout	946/376 UofC	1	99.3	4.2	81.1
			5	80.9	48.9	60.9
			10	63.8	85.1	80.6
Jiménez-García et al. 2020 [18]	Temporal, spectral, and non-lineal features / FCBF / Adaboost / - / Bootstrap & Holdout	974/390 UofC	1	99.4	1.3	80.5
			5	62.3	63.1	62.8
			10	39.8	89.6	79.0
Barroso-García et al. 2021 [16]	Bispectral features / FCBF / MLP / - / Bootstrap & Holdout	946/376 UofC	1	94.1	11.2	78.1
			5	78.7	50.6	61.2
			10	55.9	83.2	77.4
Barroso-García et al. 2021 [17]	Wavelet features / FCBF / Adaboost / - / Bootstrap & Holdout	946/376 UofC	1	79.9	47.2	73.6
			5	74.4	47.2	57.5
			10	41.1	85.5	76.1
	Wavelet features / FCBF / BY-MLP / - / Bootstrap & Holdout		1	100.0	0.0	80.9
			5	77.3	45.1	57.1
			10	50.0	76.0	70.5
Our proposal	- / - / CNN / Grad-CAM & SHAP / Holdout	1,638/306 CHAT	1	92.5	44.8	82.0
			5	63.7	95.8	86.3
			10	61.9	97.4	92.5
		515/103 CCSHS	1	66.7	74.1	70.9
			5	50.0	100.0	97.1
			10	66.7	100.0	99.0
		974/390 UofC	1	87.3	52.0	80.5
			5	57.5	95.5	81.3
			10	54.2	97.4	88.2
		545/218 UofT	1	92.2	41.6	74.3
			5	78.7	87.9	85.3
			10	72.7	93.5	90.4

Acc = Accuracy; BY-MLP = Multilayer perceptron neural network with Bayesian approach; CCSHS = Cleveland Children’s Sleep and Health Study; CHAT = Childhood Adenotonsillectomy Trial; CNN = Convolutional neural network; e/h = events/hour; FCBF = Fast correlation-based filter; FSLR = Forward stepwise logistic regression; Grad-CAM = Gradient-weighted Class Activation Mapping; LR = Logistic regression; MLP = Multilayer perceptron neural network; Se = Sensitivity; SHAP = Shapley Additive Explanations; Sp = Specificity; UofC = University of Chicago; UofT = University of Tennessee; XAI = Explainable Artificial Intelligence.

One of the most notable aspects of our model was its ability to consistently achieve high diagnostic accuracy across all analyzed datasets. This revealed the strength of airflow-based analysis in capturing more complex patterns in the pathophysiology of pediatric OSA. Moreover, the inclusion of interpretability techniques such as Grad-CAM and SHAP added significant value that was not present in the other compared studies. These tools would not only help to understand which parts of the airflow signal the model focused on when making its predictions but could also facilitate validation of the CNN-based decisions to clinicians. This transparency in decision-making would be a step forward in the adoption of DL models in clinical settings, where confidence in the predictions of the model is key to their implementation.

As a result, our approach not only offers substantial improvements in diagnostic performance but also presents a more adaptable and comprehensible methodology, positioning it as a valuable and powerful tool for the early and accurate diagnosis of pediatric OSA. Additionally, it was validated with a larger number of subjects (1,017 subjects compared to ranges of 253 to 935 in other studies [22], [23], [24], [25], [46]) from four independent datasets, which enhanced the generalization capability of our model with respect to others and reinforced its applicability in broader and more diverse clinical contexts.

### F. Limitations and Future Work

Despite the promising results obtained with our airflow-based explainable CNN for the automatic diagnosis of pediatric OSA, it is essential to consider some limitations that could influence the interpretation of our findings. Although 3,672 recordings from four independent datasets were used, the CNN model was trained exclusively with CHAT and CCSHS, as the airflow recordings from UofC and UofT did not include annotations regarding the onset and offset of apneic and hypopneic events. In addition, heterogeneity was observed across these datasets. Although detailed technical information about sensors, configurations, or acquisition settings was not available, we observed statistically significant differences in sociodemographic and clinical variables between these datasets (not within the training, validation, and test subsets of each). These differences may have contributed to the slight reduction in generalization capability observed in the independent test sets (UofC and UofT). Despite this, our CNN model achieved higher diagnostic performance than classical FE approaches based on pediatric airflow from the UofC dataset. Future studies should address this issue through data harmonization, signal normalization, and multicenter training strategies, as well as by incorporating airflow recordings from diverse pediatric populations characterized by different sociodemographic and clinical factors. Such efforts will enable a more robust and representative evaluation of the proposed model and enhance its clinical applicability.

Another important challenge identified in our study was the lower performance in the classification of moderate OSA cases, which showed lower sensitivity and greater overlap with adjacent categories. This limitation reflects both the clinical heterogeneity of this group, often representing a transitional stage between mild and severe OSA, and its status as a minority class in our cohort (996 without OSA, 1610 mild, 524 moderate, 542 severe), while the majority class across all cohorts was mild OSA. This imbalance may bias DL approaches toward favoring majority classes, reducing accuracy in minority groups. This limitation is not unique to our model, as similar difficulties in the classification of moderate OSA cases have also been reported in previous works [22], [23], [24], [25]. These studies likewise highlighted that moderate OSA is a clinically heterogeneous and less distinct category. Thus, an interesting direction for future research would be to explore data-balancing strategies (e.g., SMOTE or stratified sampling), algorithmic approaches (e.g., weighted or focal loss) and model-level solutions (e.g., ensemble classifiers or hybrid architectures) to improve the moderate OSA classification. Such approaches could enhance the discriminative capacity of explainable CNNs in this clinically complex group.

Although the use of a single sensor simplifies considerably the OSA detection process, it may limit the ability of the model to fully capture the complexity of OSA, particularly in children with specific characteristics or comorbidities. Therefore, it would be valuable to explore the implementation of models that integrate airflow signals from multiple sensors simultaneously, such as thermistors and pressure probes, which could enhance the accuracy of automatic diagnosis.

Furthermore, no signal quality indices (SQIs) or exclusion thresholds were applied during preprocessing. While this avoided discarding potentially valuable information and preserved the inherent variability of multicenter data, this choice also entails a limitation. Future research should incorporate SQIs, adaptive artefact detection methods, automatic artifact-screening algorithms, or even DL models specifically trained to detect noisy airflow segments, in order to improve robustness and ensure reliable deployment in real-world clinical settings.

It is also important to note that the study was conducted using retrospective recordings. The lack of external evaluation in real-world conditions, i.e., in home environments or diverse clinical settings, could limit the generalizability of these results. Therefore, conducting validation studies in multiple clinical environments and carrying out trials with prospective home-based recordings to confirm the effectiveness of our CNN model are warranted in the future. The next steps should include validation in real-world, multicenter environments with prospectively collected home-based airflow signals. Finally, the deployment of a system prototype in an operational clinical environment will allow a comprehensive evaluation of the tool, its integration into the workflows of the sleep unit, and its comparison against the PSG gold standard. As a future direction, it would also be interesting to use the developed explainable model for long-term monitoring of pediatric patients. This would help to assess how the model truly impacts OSA management and long-term health outcomes in children.

Another important step toward clinical translation will be the development of a user-friendly clinical interface or dashboard that integrates the outputs of Grad-CAM and SHAP in a format adapted to medical workflows. While both techniques provide complementary information, SHAP visualizations in particular may introduce a level of detail that risks overwhelming clinical users. Future work should therefore focus on tailoring these interpretability outputs to different user profiles, e.g., presenting Grad-CAM highlights as the primary visualization for clinicians, while reserving SHAP for more in-depth analysis by researchers or sleep specialists. In addition, along with the automatic diagnosis (i.e., estimated AHI), the system would report protocol-based clinical recommendations and display model-derived annotations over the airflow signal. All this information would be integrated into the graphical interface of a desktop application, with the possibility of exporting a clinical report. Developing such an explanatory interface will be crucial to ensure that interpretability translates into real diagnostic utility and enhances physician confidence in AI-assisted pediatric OSA screening.

Finally, although interpretability techniques such as Grad-CAM and SHAP were incorporated, understanding the model’s decisions may still be complex, potentially affecting confidence in its clinical implementation. Developing educational tools and training programs could enhance the comprehension of the airflow-based explainable CNN, thereby increasing its reliability. This, in turn, could help harness the full potential of our CNN, maximizing its impact on pediatric clinical practice. Therefore, our findings not only contribute from a technical perspective but also represent a step forward in the effective integration of explainable DL approaches into pediatric clinical practice, aligning with the mission of translational medicine.

## VI. Conclusion

The proposed CNN model demonstrated high performance in estimating OSA severity in the pediatric population, significantly improving the outcomes obtained through FE techniques. The combined use of airflow signals and advanced DL methods enabled accurate classification of patients into the different OSA severity groups. Furthermore, key patterns in the respiratory signal were identified using Grad-CAM and SHAP. These methods revealed that the model prioritized regions of the signal where significant and sudden amplitude changes occurred, which were indicative of the apneic event presence. It also brought to light that there may be other conditions that disrupt the natural functioning of the respiratory system and, consequently, affect the airflow signal morphology and characteristics. This ability to automatically identify relevant physiological patterns also highlighted the potential of our model as a robust and reliable tool for the early detection of critical OSA cases. According to these results, the application of our explainable CNN for analyzing airflow could serve as a valuable and accurate complement in the automated diagnosis of OSA. Thus, our proposal could represent a significant contribution to optimizing the diagnostic process, especially in resource-limited settings or in scenarios where minimizing reliance on traditional polysomnographic tests is desired.

## Conflicts of Interest

There are no conflicts of interest that could inappropriately influence this research work.

## Author Contributions

Data collection: E. Dayyat and D. Gozal. Medical diagnostics: E. Dayyat and D. Gozal. Study design: V. Barroso-García, F. Vaquerizo-Villar, G. C. Gutiérrez-Tobal, T. Leppänen, and R. Hornero. Implementation: V. Barroso-García and F. Vaquerizo-Villar. Data analysis: V. Barroso-García, F. Vaquerizo-Villar, G. C. Gutiérrez-Tobal, and T. Leppänen. Manuscript writing: V. Barroso-García. Manuscript review: V. Barroso-García, F. Vaquerizo-Villar, G. C. Gutiérrez-Tobal, E. Dayyat, D. Gozal, T. Leppänen, and R. Hornero. Funding acquisition: R. Hornero. All authors gave their final approval of this version of the manuscript.

## Ethical Considerations

1. **Human subject ethics review approvals or exemptions:** This work has been conducted according to the Declaration of Helsinki.
2. **Informed consent:** The informed consent was obtained from all children caretakers as part of the study protocols of CHAT (NCT00560859, defined in Marcus et al. [31]), CCSHS (established in Rosen et al. [33]), UofC (#11-0268-AM017, #09-115-B-AM031, and #IRB14-1241), and UofT (TN generated: 16-04774-XM).
3. **Privacy and confidentiality:** All data are anonymized.
4. **Compensation details:** Not applicable.

## Supplementary Materials

Supplementary Materials
