# Supplementary material for: An Explainable Deep-Learning Approach to Detect Pediatric Sleep Apnea From Single-Channel Airflow
Source: IEEE J Transl Eng Health Med. 2025 Oct 24;13:517–31. doi: 10.1109/JTEHM.2025.3625388 (PMC12772987; doi:10.1109/JTEHM.2025.3625388)
Supplement: Supplementary Materials [file supp1-3625388.docx]

TABLE S1

Demographic and clinical information of the children involved in our study.

|  | **CHAT** | **CCSHS** | **UofC** | **UofT** | ***p*-value** |
| --- | --- | --- | --- | --- | --- |
| **Subjects (*n*)** | 1638 (44.6%) | 515 (14.0%) | 974 (26.5%) | 545 (14.9%) | -- |
| **Age (years)** | 7.0 [2.0] ^a, b^ | 17.7 [0.5] ^a, d, e^ | 6.0 [6.0] ^b, d, f^ | 7.2 [7.6] ^e, f^ | 5.93 · 10^-300^ |
| **Males (*n*)** | 761 (46.5%) ^b^ | 260 (50.5%) ^d^ | 599 (61.5%) ^b, d^ | 293 (53.8%) | 6.80 · 10^-109^ |
| **BMI (kg/m^2^)** | 17.3 [6.2] ^a, c^ | 23.3 [6.2] ^a, d, e^ | 17.8 [6.1] ^d, f^ | 19.5 [12.1] ^c, e, f^ | 5.54 · 10^-108^ |
| **AHI (e/h)** | 2.5 [4.8] ^a, b^ | 0.8 [1.5] ^a, d, e^ | 3.8 [7.8] ^b, d, f^ | 2.3 [5.8] ^e, f^ | 2.01 · 10^-87^ |
| **No OSA (*n*)** | 355 (21.7%) ^a, c^ | 294 (57.1%) ^a, d, e^ | 171 (17.5%) ^d, f^ | 176 (32.3%) ^c, e, f^ | 4.13 · 10^-67^ |
| **Mild OSA (*n*)** | 812 (49.6%) ^a, b, c^ | 193 (37.5%) ^a^ | 398 (40.9%) ^b^ | 207 (38.0%) ^c^ | 5.28 · 10^-09^ |
| **Moderate OSA (*n*)** | 253 (15.4%) ^a^ | 16 (3.1%) ^a, d, e^ | 176 (18.1%) ^d^ | 79 (14.5%) ^e^ | 3.34 · 10^-14^ |
| **Severe OSA (*n*)** | 218 (13.3%) ^a, b^ | 12 (2.3%) ^a, d, e^ | 229 (23.5%) ^b, d, f^ | 83 (15.2%) ^e, f^ | 5.39 · 10^-27^ |

Data presented as median [interquartile range] or n (%). BMI = body mass index; AHI = apnea-hypopnea index; e/h = events/hour; OSA = obstructive sleep apnea; CHAT = Childhood Adenotonsillectomy Trial; CCSHS: Cleveland Children's Sleep and Health Study; UofC = dataset from the University of Chicago; UofT = dataset from the University of Tennessee. *p*-values for multiple comparisons obtained by means of Kruskal–Wallis for continuous variables and Chi-square for categorical variables. Pairwise differences were evaluated using post-hoc Mann–Whitney U test and Fisher test with Bonferroni correction: ^a^ Significant differences (*p*-value < 0.05) between CHAT and CCSHS; ^b^ Significant differences (*p*-value < 0.05) between CHAT and UofC; ^c^ Significant differences (*p*-value < 0.05) between CHAT and UofT; ^d^ Significant differences (*p*-value < 0.05) between CCSHS and UofC; ^e^ Significant differences (*p*-value < 0.05) between CCSHS and UofT; ^f^ Significant differences (*p*-value < 0.05) between UofC and UofT;

TABLE S2

Performance obtained in k terms for each of the convolutional neural network (CNN) configurations evaluated in the validation stage.

| **Segment size** | ***N_c_*** | ***r_drop_*** | ***k*** |
| --- | --- | --- | --- |
| 5 | 6 | 0.0 | 0.3191 |
| 5 | 6 | 0.1 | 0.2226 |
| 5 | 6 | 0.2 | 0.3660 |
| 5 | 7 | 0.0 | 0.3328 |
| 5 | 7 | 0.1 | 0.3402 |
| 5 | 7 | 0.2 | 0.2463 |
| 5 | 8 | 0.0 | 0.3389 |
| 5 | 8 | 0.1 | 0.3164 |
| 5 | 8 | 0.2 | 0.3443 |
| 10 | 6 | 0.0 | 0.3382 |
| 10 | 6 | 0.1 | 0.3214 |
| 10 | 6 | 0.2 | 0.3032 |
| 10 | 7 | 0.0 | 0.3251 |
| **10** | **7** | **0.1** | **0.3688** |
| 10 | 7 | 0.2 | 0.2785 |
| 10 | 8 | 0.0 | 0.3283 |
| 10 | 8 | 0.1 | 0.3529 |
| 10 | 8 | 0.2 | 0.2770 |
| 20 | 6 | 0.0 | 0.2996 |
| 20 | 6 | 0.1 | 0.3270 |
| 20 | 6 | 0.2 | 0.2975 |
| 20 | 7 | 0.0 | 0.3141 |
| 20 | 7 | 0.1 | 0.2180 |
| 20 | 7 | 0.2 | 0.3197 |
| 20 | 8 | 0.0 | 0.3095 |
| 20 | 8 | 0.1 | 0.3281 |
| 20 | 8 | 0.2 | 0.3246 |

*k* = Cohen's kappa; *N_c_* = number of convolutional blocks; *r_drop_* = dropout rate.


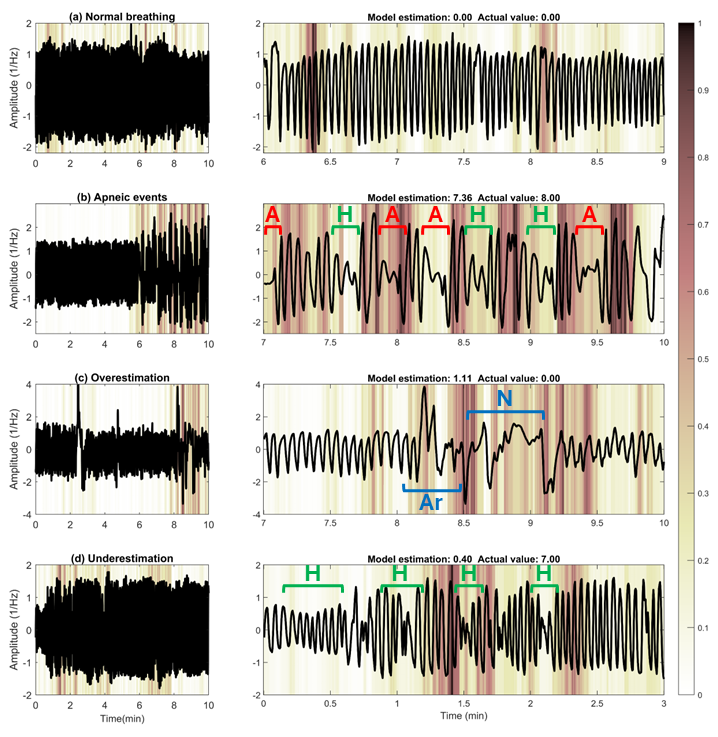


Fig. S1. Gradient-weighted class activation mapping (Grad-CAM) heatmaps generated from 10-min segments of (a) normal breathing, (b) apneic [A] and hypopneic [H] events, (c) overestimation with noise artifact [N] and arousal [Ar] events, and (d) underestimation with hypopneic [H] events scored by specialist physicians in the Cleveland Children's Sleep and Health Study (CCSHS) dataset.


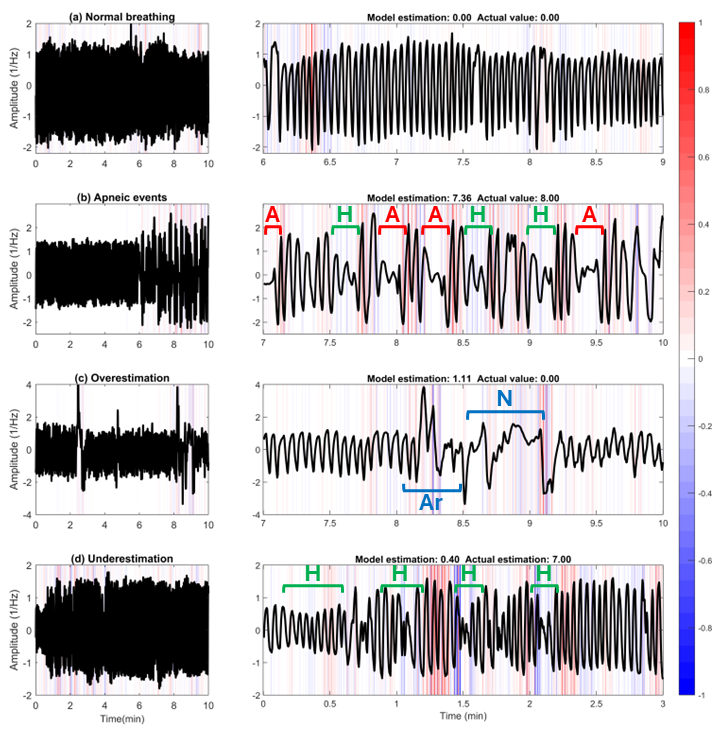


Fig. S2. Shapley additive explanation (SHAP) plots generated from 10-min segments of (a) normal breathing, (b) apneic [A] and hypopneic [H] events, (c) overestimation with noise artifact [N] and arousal [Ar] events, and (d) underestimation with hypopneic [H] events scored by specialist physicians in the Cleveland Children's Sleep and Health Study (CCSHS) dataset.
